# In which context and for whom can interventions improve leadership of surgical trainees, surgeons and surgical teams and why: a realist review protocol

**DOI:** 10.3310/nihropenres.13364.1

**Published:** 2023-03-31

**Authors:** Julia Gauly, Rachel Court, Kate Seers, Graeme Currie, Amy Grove

**Affiliations:** 1 Warwick Medical School, University of Warwick, Coventry, CV4 7AL, UK; 2 Warwick Business School, University of Warwick, Coventry, CV4 7AL, UK

**Keywords:** Leadership; surgery; realist review; distributed leadership; leadership configurations; healthcare; protocol

## Abstract

**Background:**

Improving effective leadership of individuals, groups, and healthcare organisations is essential for improving surgical performance and indirectly improving health outcomes for patients. Numerous systematic reviews have been conducted which seek to determine the effectiveness of specific leadership interventions across a range of disciplines and healthcare outcomes. The purpose of this realist review is to systematically synthesise the literature which examines in which context and for whom leadership interventions improve leadership of surgeons, surgical teams, and trainees.

**Methods:**

Several approaches will be used to iteratively search the scientific and grey literature to identify relevant evidence. Selected articles will inform the development of a programme theory that seeks to explain in which context and for whom interventions can improve leadership of surgical trainees, surgeons, and surgical teams. Next, empirical studies will be searched systematically in order to test and, where necessary, refine the theory. Once theoretical saturation has been achieved, recommendations for advancing leadership in surgery will be developed. Stakeholder and patient and public consultations will contribute to the development of the programme theory. The review will be written up according to the Realist And Meta-narrative Evidence Synthesis: Evolving Standards publication standards. No ethical review will be required for the conduct of this realist review.

**Discussion:**

The knowledge gained from this review will provide evidence-based guidance for those planning or designing leadership interventions in surgery. The recommendations will help policymakers, educationalists, healthcare providers, and those delivering or planning leadership development programmes across the surgical disciplines to design interventions that are acceptable to the surgical community and successful in improving surgical leadership.

PROSPERO registration: CRD42021230709

## Plain English summary

How do leadership development activities need to be designed in order to improve the leadership of surgeons, surgical teams and surgical trainees?

Leadership is seen to be an important skill for those working in healthcare. Healthcare systems therefore, invest a lot of money into the development of the leadership of surgeons, surgical teams, and surgical trainees. Leadership development activities include leadership courses and programmes, mentoring and coaching, feedback activities, and simulation training. To date there is no agreement on what makes leadership development activities effective or not. We also do not know whether they work for certain people or professionals more than others. It is important to find out what interventions are best, in order to spend money on leadership development effectively.

This protocol describes our plan to develop a theory explaining in which context and for whom leadership development activities work and why. We will develop the theory based on the existing literature and through discussions with experts in the field.

To make the results more reliable, we will search databases systematically and the different stages of the review will be checked by two people.

Results will feed into further research where we collect 'real world' data on leadership development activities that take place in the National Health Service (NHS) and whether they work and why. Our study will also provide guidance for those who are planning or designing leadership development activities for surgeons, surgical teams and surgical trainees.

## Introduction

### Clinical leadership in surgery

Leadership in healthcare is vital for maintaining and improving team effectiveness, clinical and financial performance, patient safety and quality ( Lyons *et al.*, 2021). Although healthcare systems invest significant resources in developing the leadership of healthcare professionals ( West *et al.*, 2015), there is no agreement on how to develop good leadership and achieving effective leadership processes remains a challenge in many areas of healthcare delivery, including surgery ( Lega *et al.*, 2017). The academic literature increasingly recognises that healthcare leadership is a shared, complex social dynamic - rather than something exclusively held by an individual person ( Lega *et al.*, 2017). However, in healthcare practice, the term leadership development is often used to describe efforts which seek to develop the skills of individuals, rather than build leadership capacity across an organisation ( Frich *et al.*, 2015).

In surgery, interventions designed to improve nontechnical surgical skills and processes (including leadership) have started to emerge in the operation room ( Hull *et al.*, 2012). Previous systematic reviews suggest that the advancing of nontechnical skills in the operating room can improve team work, performance and safety within the smaller professional groups ( Arora *et al.*, 2010; Hull *et al.*, 2012; Yule *et al.*, 2006). In the existing literature on leadership in surgery, important attributes of surgical leaders ( Patel *et al.*, 2010) and the ways that surgeons can improve their leadership skills have been identified as important for improving surgical practice and patient outcomes ( Maykel, 2013). However, focusing on this individualistic and attribute and skills focused explanation of surgical leadership limits our understanding about how leadership in the surgical profession develops across the profession, and the mechanisms and contexts which can influence and advance leadership effectiveness in the organistion ( Grove *et al.*, 2020).

Surgical leadership is not always restricted to those in formal leadership roles, for example those referred to as a Surgical Director. Leadership can be shared amongst all those involved in the delivery of care ( Harris & DeFlaminis, 2016). Hu and colleagues (2016) described how “interpersonal dynamics are highly important to operative performance” ( Hu *et al.*, 2016, p. 2). This suggests that improvement in patient outcomes after surgery are not only dependent on one individual leader (e.g., one individual surgeon), but dependent on all those who interact in the process ( The Royal College of Surgeons of England, 2014). Hence, important characteristics such as accountability and empowerment can be distributed across the surgical team. This concept of distributed leadership emerged in the early 2000s from several organisational scientists, most importantly the theory of distributed cognition and the activity theory ( Currie & Lockett, 2011; Gronn, 2000; Gronn, 2009).

Leadership in healthcare demands that up-to-date evidence is implemented into practice in order to achieve desirable patient outcomes, increased patient safety and improved quality of life ( Darzi, 2009). However, there are challenges to the use of evidence in surgical practice ( Grove *et al.*, 2018; Grove *et al.*, 2020) and the surgical specialties are often alleged to be lagging behind evidence-based practice in comparison to their medical colleagues ( Meshikhes, 2015). Consequently, the reported delays of research evidence reaching clinical practice may be compounded in the surgical specialties ( Green *et al.*, 2009; Trochim, 2010; Westfall *et al.*, 2007).

In this review, we seek to identify and understand the different types of clinical leadership which have been characterised in previous surgical research. We bring together the concepts of leadership and evidence-based practice to understand how mechanisms and contexts of healthcare organisations influence surgical leadership, and the organisational processes which can support and advance leadership in surgery.

### The need to adopt a realist approach

Since leadership interventions can be considered as complex ( Grove *et al.*, 2020), a realist review approach was deemed to be more appropriate than a traditional systematic review. Using a realist review approach, we seek to understand and develop recommendations on how, which, to what extent and in which context interventions can effectively support the development of leadership of surgeons, surgical teams and surgical trainees.

While numerous systematic reviews have been conducted to determine the effectiveness of specific leadership interventions in healthcare settings ( Davis *et al.*, 1995; De Brún & McAuliffe, 2020; Lyons *et al.*, 2021; Rosenman *et al.*, 2014; Sun *et al.*, 2018; Wong & Cummings, 2007) (for example, in medicine and nursing), a systematic synthesis of the literature to examine in which context and for whom interventions can improve the leadership of surgical trainees, surgeons and surgical teams, has not been undertaken. We aim to fill this gap by conducting a realist review.

A realist review is a theory-driven, interpretive approach to synthesise research evidence ( Brennan *et al.*, 2014), which may be qualitative, quantitative, or mixed methods ( Wong *et al.*, 2015). A key distinction between realist reviews and other review types, is that realist reviews achieve more than evaluate the effectiveness of interventions (i.e., what type of leadership development works in surgery?). Instead, realist reviews focus on understanding the interaction between context, mechanism (underlying processes or social structures) and outcomes by which an intervention, such as leadership development, can be advanced. Realist reviews set out to determine why, how, and in which context interventions work ( Paré *et al.*, 2015). Therefore, contributing to both our empirical understanding of, and the theoretical developments within, surgical leadership. In our study, we seek to combine theoretical understandings and empirical evidence to explain the relationship between the context in which leadership was applied in surgery, the mechanisms by which it worked and the outcomes that were achieved.

In order to allow for explanation building, ‘middle range’ realist programme theories, which involve “abstraction but are close enough to observed data to be incorporated in propositions that permit empirical testing” ( Merton, 1967), are developed as part of a realist review. From a realist perspective, causation is generative, meaning that interventions alter context, which then triggers mechanisms, which then produce both intended and unintended outcomes ( Wong *et al.*, 2013). Realist reviews therefore, can help to understand “how interventions work and under what circumstances the mechanisms connected to beneficial outcomes may be triggered” ( Rycroft-Malone *et al.*, 2016). Hence, this approach addresses complexity and non-linear casual relationships and therefore, is well suited to examining complex social leadership interventions in surgery.

This realist review is conducted as part of a longitudinal mixed-method study exploring how leadership and the implementation of evidence-based practice in surgery can be advanced ( Grove *et al.*, 2020). The findings will inform the conduct of semi-structured interviews with surgeons and their professional networks, to explore how surgeons learn about leadership configurations and best practice ( Grove *et al.*, 2020).

### Research questions

The research question of the realist review is:

- In which context and for whom can interventions improve leadership of surgical trainees, surgeons, and surgical teams and why?

The objectives of this realist review are:

1) To develop an initial programme theory or initial programme theories to explain in which context and for whom interventions can improve leadership of surgical trainees, surgeons and surgical teams and why
2) To test and refine the initial programme theory or programme theories
3) Based on the programme theory or theories and the review findings, to develop recommendations for policymakers, researchers and practitioners
4) To disseminate the realist review findings and the recommendations developed.

## Methods

**Figure 1.**
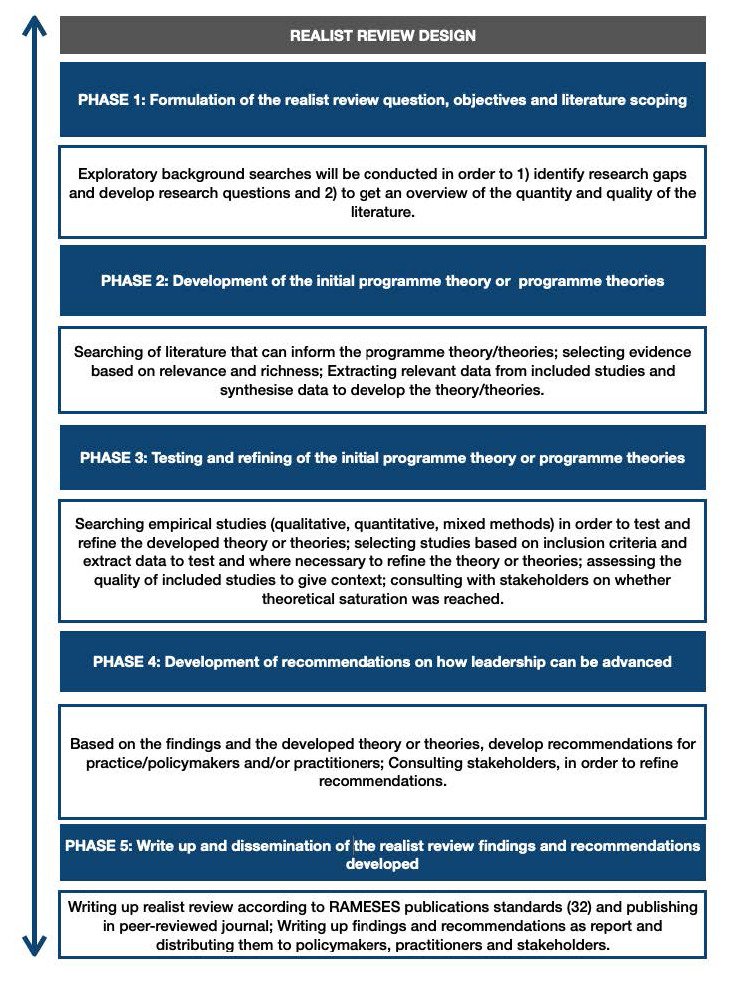
Realist Review Design.

**Figure 2.**
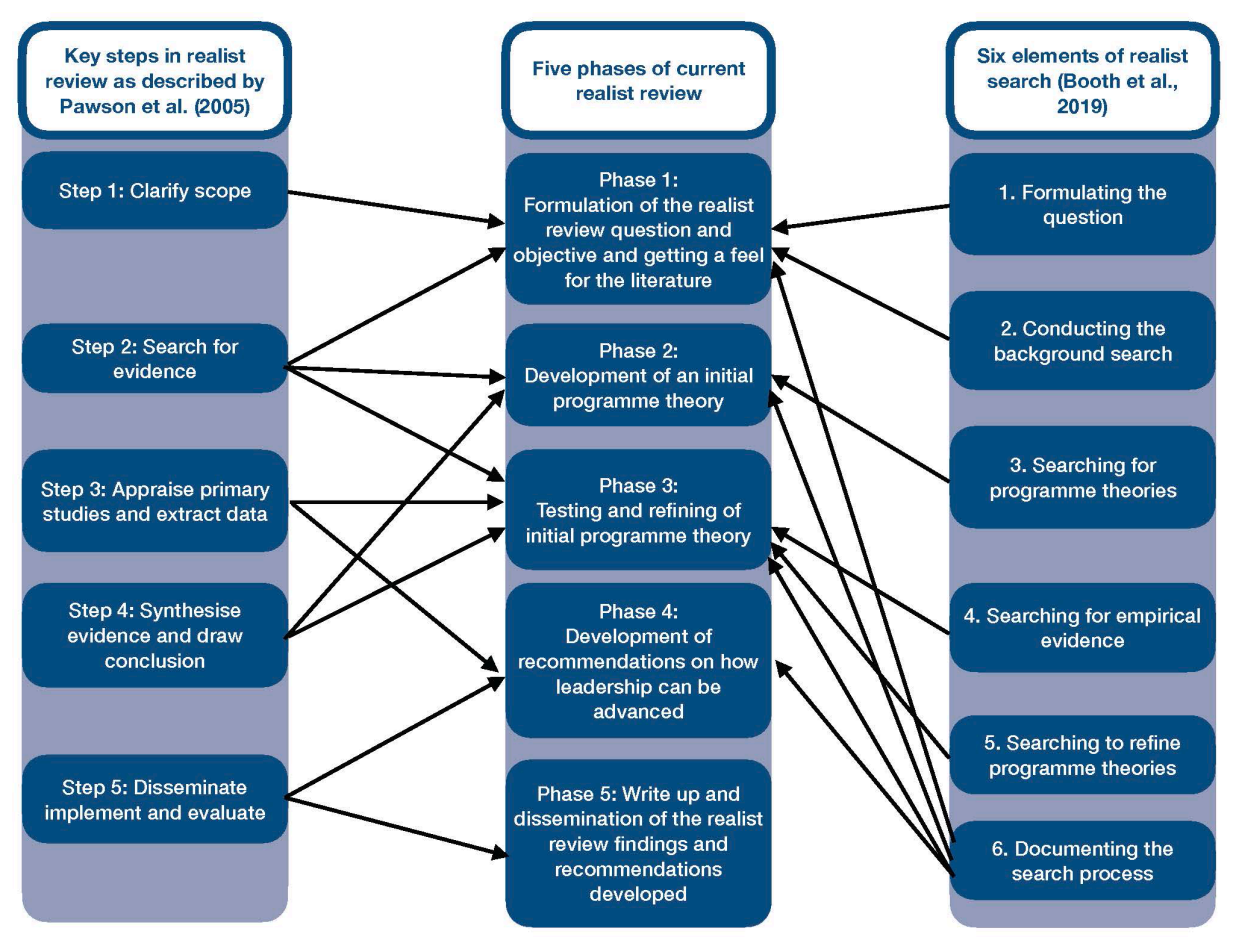
Phases of realist review.

For the purpose of this protocol, we have separated the process of the review into five phases (see Figure 1). However, we recognise that these processes are closely related and that the discrete steps of a realist review are iterative and not linear. The phases of our realist review design were informed by the realist review five steps described Pawson *et al.* (2005) and the six elements of realist review search by Booth and colleagues (2019) ( Booth *et al.*, 2019; Pawson *et al.*, 2005). Figure 2 presents which phases of this realist review were informed by the methods outlined by Pawson *et al.,* and Booth *et al.,*

In contrast to systematic reviews, in which typically only one single literature search is conducted to answer a specific research question, the realist approach uses multiple searches conducted iteratively throughout the review process ( Booth *et al.*, 2019; Pawson *et al.*, 2005). In our realist review, at least three literature searches will be conducted as part of phases two and three. As suggested by Pawson and colleagues ( Pawson, 2006), our review team contains a senior information specialist (RC) who will be involved in all stages of the review, and contribute significantly to phases 2 and 3. Information specialists are experts in searching and documenting searches and have valuable knowledge to contribute to the iterative process of searching that is needed in a realist review ( Booth *et al.,* 2019).

Stakeholder involvement is also vital for the identification of relevant literature ( Pawson *et al.*, 2004) and the validation and refinement of developing theory ( Pawson *et al.*, 2004). Consultation with experts in the field of leadership and surgery will also provide a reality check as to whether findings are consistent with experience and knowledge from practice ( Brennan *et al.*, 2014). A national group of stakeholders has been convened to support this review as it progresses, including NHS clinicians, academics, and a larger group of patient and public contributors. As suggested, the realist review process is iterative, meaning that changes may occur, and phases may be conducted repeatedly or in parallel to each other rather than sequentially. Any changes made to the research protocol, which was prepared using the PRISMA-P checklist and the RAMSES checklist ( Grove, 2023b; Grove, 2023c), will be documented as necessary in the final study report. The five phases of our review will now be described in detail.

### Phase 1: Formulation of the realist review question, objectives and literature scoping

Most structured literature reviews require reviewers to formulate a focused research question and begin to scope the literature ( Booth *et al.*, 2019; Pawson *et al.*, 2005). This is also true for this realist review and the aim of phase 1. In order to achieve phase 1 and to develop this review protocol, exploratory background searches were conducted by two reviewers (JG, AG) and gaps in the literature were identified. Search terms related to ‘leadership’ and ‘surgery’ were used during the exploratory background search (see underlying data – Grove, 2023a). Through discussion with the wider research team, the research question and objectives were developed (see 1. Introduction). The first research objective will be addressed in phase 2. The second research objective will be addressed in phase 3. The third research objective will be addressed in phase 4. The exploratory background searches did not follow any specific technical or procedural rules ( Pawson, 2006), however, they allowed us to begin to explore the quantity and quality of the surgical leadership literature.

### Phase 2: Development of an initial programme theory or programme theories

In phase 2, the first research objective of this realist review, which is ‘to develop an initial programme theory or programme theories to explain how, to what extent and in which context leadership in surgery can be influenced’ will be addressed.

***Literature search*.** Searching for evidence that can inform the programme theory can be challenging, particularly because studies that include theories rarely include terms such as ‘theory’ in their titles. Therefore, diverse approaches to literature searching will be taken including searches of a range of bibliographic databases, using search filters where necessary in larger databases, and using techniques such as citation pearl growing, forward citation searching (using Web of Science and Scopus), and cluster searching to identify further relevant articles ( Academic Unit of Health Economics, 2018; Booth *et al.*, 2013). We will search for literature related to leadership in surgery but may also draw on literature from different but related fields, including organisational and implementation science.

Additionally, Google (using the advanced search feature) and several healthcare websites will be searched or browsed to identify relevant grey literature (including NHS evidence, The Kings Fund, The Royal College of Surgeons of England, Nuffield Trust, NHS England/NHS Improvement, Institute for Healthcare Improvement, The Leadership Academy, Skills for Care, King's Fund, Advance HE, The Institute of Healthcare Management, Faculty of Medical Leadership and Management). Search terms will include words around theory (e.g., ‘theory’, ‘programme’, ‘model’, ‘logic model’, and ‘framework’) as well as content terms such as ‘leadership’ and/or ‘surgery’ or ‘healthcare’. All records identified in bibliographic databases will be uploaded into EndNote software and deduplicated. Grey literature results from websites will be screened by two reviewers (JG, AG) online and relevant documents added to EndNote. The reference list of all included documents will be screened for potentially relevant documents.

Evidence will be searched without date restrictions and publication types will include letters, editorials and reviews. Only documents in English will be included in this review due to limited translation resources. We will contact our stakeholder group to request additional documents which they believe may be relevant for the development of an initial programme theory or programme theories.

***Selecting evidence*.** The lead reviewer (JG) will initially filter the documents according to their titles and abstracts. Subsequently, full texts of documents that were found at title and abstract stage to be potentially relevant will be retrieved. All of those full texts will then be reviewed by the lead reviewer and evidence will be selected according to its relevance and richness. We seek to understand whether the evidence will help explain how, to what extent and in which context leadership appears to influence surgical practice. A second reviewer (AG) will independently review at least 20% of documents reviewed by the lead reviewer at both the title and abstract and the full text stages. While reviewing the documents, the reviewers will highlight relevant parts in the documents and take notes and make comments on whether the documents can inform the initial programme theory or programme theories. According to our initial discussions, the reviewers will decide whether or not to include a document. Any disagreement that cannot be resolved will be checked by a third reviewer (KS).

***Data extraction*.** The lead reviewer will then extract relevant information from all included documents, which explains how, to what extent, and in which context leadership in surgery can be influenced. Extracted information may be mapped onto a context (where does intervention occur and who initiates intervention), intervention (interventions, strategies or processes that influence leadership), mechanism (actions taken) or outcomes (unintended or intended results). The second reviewer will check at least 20% of all extracted data for accuracy.

***Theory development*.** The reviewers will then review the extracted evidence and synthesise the different configurations of context, interventions, mechanisms and outcomes with regards to leadership in surgical practice. Findings will be described in words and figures, the data sources from which the initial programme theory or programme theories was or were derived from will be recorded.

***Theory refinement*.** Through discussion with the research team and stakeholder group, we will seek to identify whether or not there are any gaps in the theory. Where necessary, further searches may be conducted, and further documents considered. Any additional searches will be documented as previously described. We acknowledge that there cannot be an absolute or complete end point to analysis of the theoretical constructs ( Low, 2019). However, that does not mean that it is not important to consider theoretical saturation. We will follow the pragmatic guidance by Low (2019) to consider whether the point of theoretical saturation has been reached during phase 2 ( Low, 2019).

### Phase 3: Testing and refining of the initial programme theory or programme theories

The aim of the third phase is to address the second research objective which is ‘to test and refine the initial programme theory or programme theories’. To achieve this objective, primary studies including qualitative, quantitative and mixed method empirical research will be identified and used to test and refine the programme theories developed throughout phase 2. In phase 3, primary studies will be identified using a more systematic search of the literature.

***Literature search*.** A systematic search in several electronic databases will be conducted. These will include, but not be limited to, Ovid MEDLINE, EMBASE, and Abi/INFORM Global. The search will be adapted for each different database. The full search for Medline has been provided as underlying data ( Grove, 2023a). Additional grey literature will be searched as appropriate. For example, the websites form the Kings Fund, NHS England/NHS Improvement and the Leadership Academy will be searched. Techniques such as citation pearl growing and forward citation searching may also be used to identify further evidence. Additionally, the references of all included documents will be screened to identify further relevant documents for consideration. Further rounds of searching will be conducted where necessary.

***Inclusion criteria*.** Documents will be included based on their relevance to the review question. We seek to understand if the article can be used to test or refine the initial programme theory or theories ( Booth *et al.*, 2013). Relevance will be determined by whether the following inclusion criteria are met:

• **Study type:** all types of primary empirical studies e.g., qualitative research, quantitative research, mixed methods research.

• **Study setting:** studies in clinical settings (e.g., hospitals, specialist clinics), in academic organisations (e.g., universities) and training settings (e.g., independent training organisations).

• **Participants:** all staff involved in or influential to the delivery of surgical practice, participants may include but are not limited to surgeons, nurses, and applied health professional and surgeon’s professional networks.

• **Intervention/ activities/ processes:** all studies that give insight into any training(s), interventions, activities, processes, or strategies that are implemented or conducted in order to influence leadership in the surgical profession. For example, this may be training that aim to advance leadership skills or the development of team working skills within surgical teams. It could also include studies that evaluate interventions, activities, or processes that are implemented to influence leadership in surgery. Studies that aim to influence individuals’ or groups’ understanding of research-evidence will only be included if they give insight into whether or not the intervention influenced leadership.

• **Outcomes:** all outcomes reported in the article that are reported as outcomes of the leadership interventions, strategies, activities, or processes that are conducted. This could include patient outcomes (e.g., mortality, patient satisfaction) but also staff outcomes (e.g., empowerment, improved communication skills) and organisational outcomes (e.g., productivity, organisation performance). Outcomes will be grouped into intended and unintended, positive and negative, self-reported and not self-reported, or short-term and long-term outcomes as appropriate.

***Document selection*.** All records from bibliographic databases will be uploaded into EndNote v20 ( *Endnote 20*, no date) and their titles and abstracts screened by the lead reviewer for relevance to the inclusion criteria. The second reviewer will screen all records independently. According to the discussion between reviewers, records will be included or excluded for full text screening. Grey literature sources will be screened online by the lead reviewer. The lead reviewer will retrieve all full texts of those documents deemed potentially relevant and both reviewers will screen all articles’ full texts. Any disagreement that cannot be resolved will be checked by a third reviewer (KS). 

***Data extraction*.** In a realist review, documents are rarely used as a whole for the analysis ( Kastner *et al.*, 2011). Instead, small sections of the included documents will be used to test our preliminary programme theory or programme theories ( Brennan *et al.*, 2014; Kastner *et al.*, 2011). In contrast to traditional systematic reviews, where standardised forms are used to extract data, we will use notes and annotations to assimilate and synthesise relevant information from the included papers ( Power *et al.*, 2019; Wong *et al.*, 2015). For this review, we will adopt a hybrid approach to data extraction ( Weetman *et al.*, 2019): first of all, software such as NVivo v10 for Mac ( *NVivo*, no date) will be used to annotate data for contexts, mechanisms, and outcomes and programme theories and to manage reviewer notes. Second, data extraction forms will be developed iteratively to extract descriptive study characteristics and to categorise all included documents. Information that we expect to extract is shown in Table 1. The lead reviewer will extract data for 100% of all included documents and the second reviewer will check at least 20% of the extracted data for accuracy.

**Table 1.** Data expected to be extracted from empirical evidence as part of phase 3.

• Study design/type • Country • Limitations • Healthcare service areas in which leadership is situated (surgical speciality/hospital) • Description of leadership activities • Any reported outcomes in relation to leadership activities enabling or inhibiting contexts linked to leadership/ leadership strategies • Clarification and explanation about context, mechanism, and outcome configurations related to the research question.

***Assessment of rigour*.** All studies included to test and refine the theory or theories in phase 3 will be assessed for their rigour to determine whether the methods used to generate the relevant data are credible and trustworthy ( Brennan *et al.*, 2014). Documents will not be excluded based on their rigour, as extracts of documents with a lower rigour reporting may still have valid contributions. However, this process will be conducted to give context to the reader. As we will include qualitative, quantitative and mixed methods studies, will be use the Mixed Methods Appraisal tool (MMAT) to assess the rigour of all included studies ( Hong *et al.*, 2018). MAAT has been used in previous realist reviews to assess the quality of studies ( Bedwell *et al.*, 2017; Thapa *et al.*, 2018; Wozney *et al.*, 2017) and is well suited to assess the rigour of studies of all types of study designs. The lead reviewer will assess the rigour of all included studies and the second reviewer will assess 20% of all included studies for accuracy. Disagreement will be resolved through a third reviewer (KS). Results of the assessments of rigour will be recorded in summary tables and presented in the findings of the realist review.

***Theory testing and refinement*.** Phase 2 results in the development of an initial programme theory or theories linking outcomes with context, mechanisms and implementations. The studies included in phase 3 will then be used to test, confirm, refute, or refine the theory or theories. This will be done by analysing similarities and differences between the context, mechanism, and outcome configurations from the initial programme theories and the empirical evidence included in the phase 3. The analysis will be used to iteratively feed back into the initial programme theory or theories we developed in phase 2. Not all studies included in phase 3 may be used to test and refine theories. Instead, we will use the empirical evidence for testing until theoretical saturation has been reached ( Low, 2019). If we still identify gaps in the theory or theories, we may conduct additional searches to aim to close these gaps. All additional searches will be documented and justified using methods described in phase 3.

### Phase 4: Development of recommendations on how leadership can be advanced

Using the findings of the realist review and the theory we have developed; we will develop recommendations on how leadership can influence surgical practice. Throughout the review process, we anticipate finding gaps in the research literature. Hence, recommendations may focus on what type of additional research needs to be conducted to better understand how interventions, processes and strategies can advance surgical leadership. The theoretical understanding we develop during the review will enable us to develop clear, evidence-based recommendations for policymakers, health organisations, and practitioners on what leadership development practices should be introduced, stopped or changed in order to advance leadership in surgery.

### Phase 5: Write up and dissemination of the realist review findings and recommendations developed

The review will be written up in line with the guidance the Realist And Meta-narrative Evidence Synthesis: Evolving Standards (RAMESES) publication standards ( Wong *et al.*, 2013). We aim to publish the realist review in a peer-reviewed journal. An executive summary of the findings and recommendations of the realist review will be produced and shared with policymakers, practitioners and educationalists interested in, or responsible for surgical leadership development. Findings will also be shared with our stakeholder group who took part in the review process. Where appropriate we will disseminate the findings of the review at conferences attended by both healthcare professionals and academic audiences. As this realist review is part of a larger mixed methods project, the findings will be used to inform primary data collection for longitudinal semi-structured interviews with surgeons and their professional network(s).

## Discussion

Effective surgical leadership in is an important part of healthcare practice to improve care delivery, to ensure patient safety and effective team work ( Currie *et al.*, 2014; Giddings & Williamson, 2007; Royal College of Surgeons, 2014). However, interventions which seek to influence leadership are complex and context-sensitive ( Lega *et al.,* 2017). Therefore, leadership development programmes which are shown to work (e.g., improve health and organisational outcomes) in one area of the NHS may not be transferable across healthcare organisations, or effective in different surgical groups (e.g., surgeons at early and late career stages, surgeons of differing specialities, or gender identities). This realist review will enable a greater understanding of the mechanisms and contexts influencing leadership in the surgical profession and contribute to advancing leadership and related outcomes in surgery.

Focussing on improving or expanding technical skills is no longer sufficient to deliver modern, safe surgical care ( Agha *et al.,* 2015). Instead, those who make decisions for patients need to ensure that individual, groups and organisations partake in leadership development and obtain knowledge and processes which are appropriate and effective. We anticipate that the knowledge and information gained from this realist review can help to inform policymakers, healthcare providers and those delivering and planning leadership development on the mechanisms and context that need to be in place to advance leadership in surgery.

## Data Availability

Figshare: Additional File 1 .docx. https://doi.org/10.6084/m9.figshare.21988613.v1. ( Grove, 2023a). This project contains the following underlying data: Additional File 1 .docx. (Full search strategy for Medline). Data are available under the terms of the Creative Commons Attribution 4.0 International license (CC-BY 4.0). Figshare: PRISMA-P checklist for ‘In which context and for whom can interventions improve leadership of surgical trainees, surgeons and surgical teams and why: a realist review protocol. https://doi.org/10.6084/m9.figshare.21989780.v1. ( Grove, 2023b). Figshare: RAMSES checklist for ‘In which context and for whom can interventions improve leadership of surgical trainees, surgeons and surgical teams and why: a realist review protocol. https://doi.org/10.6084/m9.figshare.21988598.v1. ( Grove, 2023c). Data are available under the terms of the Creative Commons Attribution 4.0 International license (CC-BY 4.0).
