# Supplementary material for: In which context and for whom can interventions improve leadership of surgical trainees, surgeons and surgical teams and why: a realist review protocol
Source: NIHR Open Res. 2023 Mar 31;3:16. [Version 1] doi: 10.3310/nihropenres.13364.1 (PMC10593322; doi:10.3310/nihropenres.13364.1)
Supplement: Peer review history

# Reviewer report: Reviewer response for version 1

**DOI:** 10.3310/nihropenres.14495.r29307

**Published:** 2023-07-10

**Authors:** Sharon Marie Weldon

**Affiliations:** 1 University of Greenwich, London, England, UK

Thank you for the opportunity to review this paper. I commend the methodological approach to searching the literature which is a good pragmatic approach due to the complexity of the search and will more likely answer the question appropriately compared to other methods. 

 To improve the protocol, I would suggest that the authors include all languages, as although I appreciate they don't have the language resources readily available, this day in age there is software than can do this and often the authors themselves can send a translated copy if requested. By not including all languages, the search and its results are at risk of only making visible limited cultural approaches and therefore may miss the opportunity to truly understand leadership in different contexts and cultures.

Is the study design appropriate for the research question?

Yes

Is the rationale for, and objectives of, the study clearly described?

Yes

Are sufficient details of the methods provided to allow replication by others?

Yes

Are the datasets clearly presented in a useable and accessible format?

Not applicable

Reviewer Expertise:

Leadership, communication, simulation, all types of systematic reviews and varied methodological approaches.

I confirm that I have read this submission and believe that I have an appropriate level of expertise to confirm that it is of an acceptable scientific standard.

## References

[1] : A Video Analysis of Intra- and Interprofessional Leadership Behaviors Within "The Burns Suite": Identifying Key Leadership Models. *J Surg Educ* .2016;73(1) : 10.1016/j.jsurg.2015.09.011 31-9 10.1016/j.jsurg.2015.09.011 26699279

# Reviewer report: Reviewer response for version 1

**DOI:** 10.3310/nihropenres.14495.r29488

**Published:** 2023-07-10

**Authors:** Anurag Saxena

**Affiliations:** 1 University of Saskatchewan, Saskatoon, Saskatchewan, Canada

**Rationale and Objectives**: Rationale for review (of leadership training / education for surgical trainees) and the need for a realist approach are both well described. The former is framed against the backdrop of leadership in healthcare and leadership by physicians and its link to healthcare organizational performance and patient outcomes. Justification for the realist approach is rooted in the complex and multifaceted nature of leadership development and the lack of a clear conceptual framework.

**Study Design appropriate to the research question(s):** The mixed-methods approach and the six stages (Pawson’s iterative stages) form the backbone of the study design – shown in Fig 1. The authors have described this approach in sufficient detail that demonstrates the ability to conduct this study. The overall research question, “ *In which context and for whom can interventions improve leadership of surgical trainees, surgeons, and surgical teams and why?*” is best answered using the realist approach and the objectives describe 4 specific stages of the work.

**Sufficient details of the methods provided to allow replication:** The Methods section is described in considerable details in the five phases (Fig 2 ) and steps in each phase and a section on ensuring rigor of this study (both quantitative and qualitative aspects). For the literature search inclusion criteria are clearly described. Supporting theories for quality appraisal of articles and data extraction are described.

**Are the data sets clearly presented in a useable and accessible format:** The availability of data is and reporting guidelines (PRISMA-P) and well described.

Is the study design appropriate for the research question?

Yes

Is the rationale for, and objectives of, the study clearly described?

Yes

Are sufficient details of the methods provided to allow replication by others?

Yes

Are the datasets clearly presented in a useable and accessible format?

Not applicable

Reviewer Expertise:

Leadership, Leadership development, Systems and Policy in Healthcare and medical education, Organizational and Administrative sciences, Mixed methods studies, Interdisciplinary research

I confirm that I have read this submission and believe that I have an appropriate level of expertise to confirm that it is of an acceptable scientific standard.

# Reviewer report: Reviewer response for version 1

**DOI:** 10.3310/nihropenres.14495.r29152

**Published:** 2023-06-29

**Authors:** Darshini R. Ayton

**Affiliations:** 1 Department of Epidemiology and Preventive Medicine, School of Public Health and Preventive Medicine, Monash University, Clayton, Victoria, Australia

This article is a protocol for a realist review which aims to identify and understand interventions to improve clinical leadership in surgery taking into consideration contextual factors and organizational support. Based on the review and discussions with experts, the authors will develop a theory of leadership for surgical trainees, surgeons, and teams to explain the context and for whom leadership interventions work and why. 

- The article is very well written.
- The rationale for adopting a realist approach to this review is well argued and explained.
- A strength of this review is that it is being conducted alongside primary data collection and hence this data collection will be informed by the findings of the review.
- The phases of the review are well described.
- The use of the MMAT includes a justification that it is relevant for all study designs. 
- Review search terms are available.

Is the study design appropriate for the research question?

Yes

Is the rationale for, and objectives of, the study clearly described?

Yes

Are sufficient details of the methods provided to allow replication by others?

Yes

Are the datasets clearly presented in a useable and accessible format?

Not applicable

Reviewer Expertise:

Implementation science, health services research, aged care, dementia

I confirm that I have read this submission and believe that I have an appropriate level of expertise to confirm that it is of an acceptable scientific standard.

# Reviewer report: Reviewer response for version 1

**DOI:** 10.3310/nihropenres.14495.r29485

**Published:** 2023-06-29

**Authors:** Abi Sriharan

**Affiliations:** 1 University of Toronto, Toronto, Ontario, Canada

This study aims to use a realist review to gather and analyze literature on how leadership interventions can improve the leadership skills of surgeons, surgical teams, and trainees. The study will focus on identifying the contexts and groups where these interventions have been effective.

 This research on leadership programs for surgical specialties is grounded in the idea that healthcare leadership is a multifaceted and intricate social phenomenon. The protocol's realist approach is an excellent fit for addressing the research question due to the complex nature of leadership interventions. The detailed description of the realist methods is helpful.

 Per the study protocol, the team has completed the initial phase of exploring pertinent literature before submitting the protocol. The methods discussed in this study's implementation are feasible due to the team's composition and the systematic approach adopted.

 To enhance this protocol, it would be beneficial to implement the following actions:

 The background/methods section should provide a more precise definition of leadership to enable a targeted literature search and data analysis. Leadership can be analyzed through different lenses, such as task, process, people, and context leadership. Researchers must determine whether they want to encompass all aspects of leadership or primarily focus on clinical leadership in the context of evidence-based practice. Such clarity will facilitate a comprehensive understanding of how healthcare organizations' mechanisms and contexts impact surgical leadership.

 Further, building on the construct of surgical leadership, consider identifying preliminary outcomes of successful surgical leadership from the literature identified in the initial scoping search. Are you interested in surgical leadership to improve surgical outcomes, team-level outcomes, patient experience-level outcomes, organizational-level outcomes etc.?

 The authors have articulated that this study is conducted as part of a longitudinal mixed-method study exploring the role of leadership in implementing evidence-based practice in surgery. Have you considered reviewing the existing theories on the implementation of EBP and leadership? As part of the preliminary theory development integrating this knowledge will be essential to construct your preliminary theory.

 In addition to collecting the data sets related to the following points:

- Study design/type
- Country
- Limitations
- Healthcare service areas in which leadership is situated (surgical specialty/hospital)
- Description of leadership activities
- Any reported outcomes concerning leadership activities enabling or inhibiting contexts linked to leadership/ leadership strategies
- Clarification and explanation about context, mechanism, and outcome configurations related to the research question

Please clarify how the data collection process will stratify data based on the leadership of surgical trainees, surgeons, and surgical teams. Would you consider collecting data based on the experiences of leaders based on their career strategy, specialty, sex and gender as well?

 Finally, you may want to refer to the following resources when defining leadership.

- Yukl, G. (1989). Managerial Leadership: A Review of Theory and Research. Journal of Management, 15(2), 251–289. https://doi.org/10.1177/014920638901500207
- Harvey, G., Kelly, J., Kitson, A., Thornton, K., & Owen, V. (2020). Leadership for evidence-based practice—Enforcing or enabling implementation? *Collegian*, *27*(1), 57-62.
- Stetler, C. B., Ritchie, J. A., Rycroft‐Malone, J., & Charns, M. P. (2014). Leadership for evidence‐based practice: strategic and functional behaviours for institutionalizing EBP. *Worldviews on Evidence‐Based Nursing*, *11*(4), 219-226.

Is the study design appropriate for the research question?

Yes

Is the rationale for, and objectives of, the study clearly described?

Yes

Are sufficient details of the methods provided to allow replication by others?

Yes

Are the datasets clearly presented in a useable and accessible format?

Not applicable

Reviewer Expertise:

Healthcare Leadership, Realist Methods

I confirm that I have read this submission and believe that I have an appropriate level of expertise to confirm that it is of an acceptable scientific standard, however I have significant reservations, as outlined above.

## References

[1] : Managerial Leadership: A Review of Theory and Research. *Journal of Management* .1989;15(2) : 10.1177/014920638901500207 251-289 10.1177/014920638901500207

[2] : Leadership for evidence-based practice—Enforcing or enabling implementation?. *Collegian* .2020;27(1) : 10.1016/j.colegn.2019.04.004 57-62 10.1016/j.colegn.2019.04.004

[3] : Leadership for evidence-based practice: strategic and functional behaviors for institutionalizing EBP. *Worldviews Evid Based Nurs* .2014;11(4) : 10.1111/wvn.12044 219-26 10.1111/wvn.12044 24986669PMC4240461
